# Benefits of Pilates in the Elderly Population: A Systematic Review and Meta-Analysis

**DOI:** 10.3390/ejihpe12030018

**Published:** 2022-02-22

**Authors:** Mário José Pereira, Rodrigo Mendes, Rui Sousa Mendes, Fernando Martins, Ricardo Gomes, José Gama, Gonçalo Dias, Maria António Castro

**Affiliations:** 1 Faculdade de Ciências do Desporto e Educação Física, Universidade de Coimbra, 3040-248 Coimbra, Portugal; 2 ESEC-UNICID-ASSERT, Instituto Politécnico de Coimbra, 3030-329 Coimbra, Portugal; a2019129465@esec.pt (R.M.); rmendes@esec.pt (R.S.M.) fmlmartins@esec.pt (F.M.); rimgomes@esec.pt (R.G.); goncalodias@fcdef.uc.pt (G.D.); 3 ROBOCORP, IIA, Instituto Politécnico de Coimbra, 3030-329 Coimbra, Portugal; maria.castro@ipleiria.pt; 4 Research Unit for Sport and Physical Activity (CIDAF) (UID/DTP/04213/2020), Universidade de Coimbra, 3040-248 Coimbra, Portugal; jgama@esec.pt; 5 Instituto de Telecomunicações (IT), 6201-001 Covilhã, Portugal; 6 CEMMPRE (UIDB/00285/2020), Universidade de Coimbra, 3030-788 Coimbra, Portugal; 7 Escola Superior de Saúde, Instituto Politécnico de Leiria, 2411-901 Leiria, Portugal

**Keywords:** elderly, health, active aging, balance, Pilates

## Abstract

The aim of this systematic review is to collect and summarize the benefits of Pilates in the elderly population (>60 years old), within the current scientific production, assessing its contribution to Healthy Ageing (HA). We used PRISMA (Preferred Reporting Items for Systematic Reviews and Meta-analysis) to select, collect, and analyse this thematic. The methodological procedures were registered in the PROSPERO database. The main results of the studies analysed (*n* = 30) point to significant differences between the intervention and the control groups in dynamic balance, strength, mobility, functional capacity, risk of falling reduction, and mental and psychological health. Thus, the results showed that Pilates may be beneficial for the health of the elderly. The meta-analysis found statistical differences between means on the dynamic balance (mean difference (MD) = −0.0, 95% CI [−0.71, −0.50]; *I*^2^: 0%) and the aerobic capacity and aerobic resistance [(MD) = 38.29, 95% CI [6.82, 69.77]; *I*^2^: 0%). Thus, it is concluded that the efficacy of Pilates has been shown in various areas of HA and has proven to be affordable and safe for the majority of people, using just a mat on the floor. Future studies should focus on the analysis of the relationship between the cost and the benefit of a Pilates intervention in the elderly population, to better understand how health costs can be minimized and to contribute to a multidisciplinary and generalized HA. Pilates has practical application for the clinicians, therapists, and health professionals that work with the elderly population.

## 1. Introduction

The number of people aged 80 years or more will triple and reach 434 million by 2050. On a world scale, the number of people aged over 60 is increasing at a yearly rate of 3%, far higher than the younger age groups. The prediction is that in 2050 the elderly will represent 22% of the population [1]. This demographic evolution has a strong social, political, and economic impact and is an indicator of the social transformation of the 21st century.

Ageing results in molecular and cellular decline, with a progressive influence on all body systems and, inherently, on the person’s psychosocial condition [2,3]. Physical activity may help reduce the speed of this decline, raising or maintaining the elder person’s intrinsic and functional capacity by improving physical capacities (e.g., strength, balance, and flexibility) [4]. Therefore, physical activity represents one of the factors that may minimize the direct influence of chronological age on the loss of bio-psychosocial function associated with ageing [5]. This multidisciplinary approach to ageing converges with the benefits of physical activity, given the pertinence and reach of its influence in the quality of life of the elderly [6]. Additionally, its efficiency and impact on the health of populations and the costs associated with these services fulfill the demands of current and future political decisions [1].

The Pilates method was developed in the 1920s by Joseph Pilates. Given its holistic approach, it is presented as one of the most efficient ways to reach the goals of Healthy Ageing (HA). It uses exercises that encompass a dualism—body and mind—which demands trunk stability, strength, and flexibility, as well as a focus on muscular control, body posture, and breathing. It uses six fundamental principles: (i) center, (ii) concentration, (iii) control, (iv) precision, (v) fluidity, and (vi) breathing. It may be done solo or in groups, with apparatuses (e.g., the Reformer or Trapezius) or on the ground (using a mat) or with only the body weight [7]. The efficiency of the Pilates method emerges from here, enabling psychomotor benefits and contributing to a better functional capacity, increasing independence and quality of life [8,9].

There has been a gradual increase in studies about the Pilates method in recent years [10]. The current research points to the efficiency of Pilates in health, particularly in physiotherapy and rehabilitation [11,12]. Additionally, there are psychological benefits [13,14] as well as benefits to the elderly person’s quality of life [15,16]. Furthermore, there is an evident economic benefit when compared with other medical procedures and the absence of relevant contraindications. However, despite this evolution only three systematic reviews analyzing the benefits of Pilates interventions for the elderly were conducted in the last 5 years.

Thus, it seems pertinent to update the question: is Pilates an effective way to promote HA? If so, how? The aim of this systematic review and meta-analysis is to collect and synthesize the benefits of Pilates in the elderly population (older than 60), assessing its contribution to HA. This review included comparative studies, where Pilates was compared with other interventions, and studies with a control group without intervention. The results of physical capacities, such as strength, flexibility, and balance, as well as psychological and well-being variables, were analysed.

## 2. Materials and Methods

### 2.1. Search Strategy

**Figure 1:**
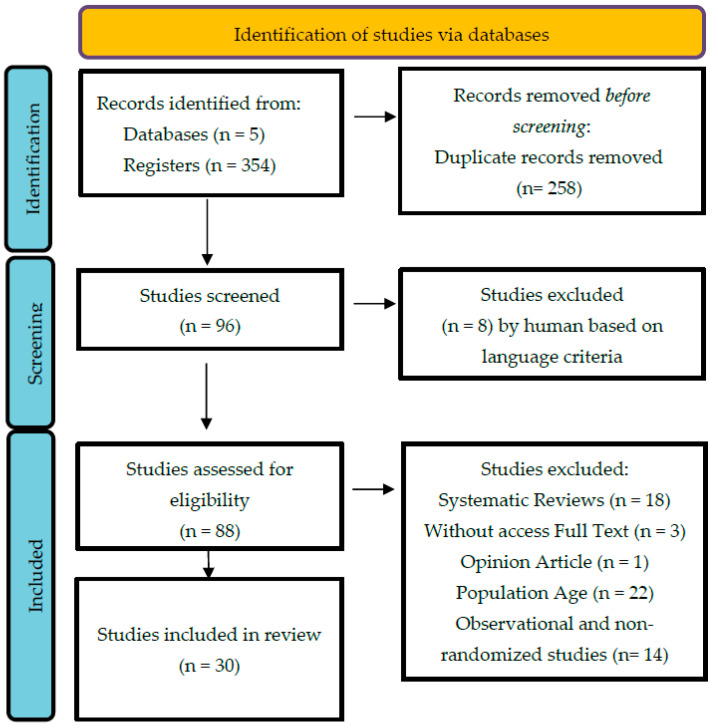
Prisma Flowchart (adapted from [18]).

This systematic review used PRISMA (Preferred Reporting Items for Systematic Reviews and Meta-analysis) [17] to select, collect, and analyse this thematic (Figure 1). The methodological procedures were registered in the PROSPERO database under the ID number CRD42021246371. Five databases were analysed: SportDiscus with Full Text; PEDro; PUBMED; Web of Science—Core Collection; and B-ON. The term “Pilates”, associated with the descriptors “elderly” or “ag*” or “old* adult” and “health” or “physical fitness” or “functional capacity”, and the Boolean operators “AND” and “OR”, were used. The timespan selection is from 2016, and it is justified with the increase in the number of published studies in this field of study, from this date, and the need to systematize and update the knowledge during this period. Afterwards, the main crossed references of the articles included in the review were analysed. No grey literature research was made, and no specialists were consulted as no valid references were found.

### 2.2. Eligibility Criteria

The inclusion criteria were the following: (i) published works between 1 January 2016 and 21 April 2021; (ii) works written in English, Portuguese, Spanish, or French; (iii) studies that used the work “Pilates” in the title or in the keywords, with a sample over 60 years of age; (iv) random clinical trials; and (v) studies where Pilates was one of the dependent variables in the experiment. The following criteria were used for exclusion: (i) publications prior to 2016; (ii) publications without full text; (iii) academic theses, books, or non-scientific articles; and (iv) studies where the Pilates method was used along with other interventions or techniques (see Figure 1).

The selection process was conducted according to the following stages: (i) research that used the descriptors in the aforementioned databases; (ii) exclusion of duplicate articles; (iii) reading of abstracts; and (iv) critical reading and assessment of the articles (cf. Figure 1). The selection and extraction of the data from the articles was conducted in two stages. Firstly, two authors (MP, RM) made an independent selection and data collection from the eligible articles. After gathering both selections, the resolution of tie situations was solved in a meeting between both authors. If needed, a third author was called to decide (MC). After this process and the reaching of a consensus higher than 85%, one of the authors (MP) completed the process for the remaining eligible articles. For a review of this nature, the most reliable source is the Random Clinical Experimental Trials (RCTs) [19]. Regardless, evidence from observational or non-randomized trials was equally included, broadening the span of the collection that could guide the intervention of the technicians using the Pilates methods in HA. To keep data quality control and the methodological requisites for this review, we chose to analyse and treat these two categories separately. Finally, the definition of an exclusion criteria of articles written in other idioms is justified by two reasons: the first is due to the fact the difficulty in assessing articles without consulting the full text would skew the data that would result from non-technical translations. The second is due to the fact that this idiom limitation has not changed the conclusions of the systematic reviews made [20].

### 2.3. Quality Assessement

PEDro (Physiotherapy Evidence Database) was used by two authors (M.J.P., R.M.) to independently register the included studies. The PEDro scale can be used in the assessment of the publication bias of the clinical trials [21]. It assesses two aspects of the quality of a clinical trial: (i) credibility, that is, internal validity and (ii) whether the article contains enough statistical information to be interpreted. The first item of the scale assesses the external validity and does not encompass the quantification of the final score. To assess the internal validity, eight criteria were used: (i) random distribution, (ii) secret allocation, (iii) comparison of groups in the beginning, (iv) blind subject, (v) therapist, (vi) evaluators, (vii) analysis by treatment intention, and (viii) complete following period (items 2–9 of the PEDro scale). To assess interpretability, statistical comparisons between the groups were used and, as reported, the (x) precision measurements and the (XI) variability (items 10 and 11 of the PEDro scale). The final score higher than 7 is attributed to a study with “high quality”. Between 5 and 6, “moderate quality” is considered. Scores lower than 4 are of “low quality”.

### 2.4. Statistical Analysis

This meta-analysis was conducted using the mean and standard deviation of the following variables: static balance, dynamic balance, balance confidence, strength and aerobic capacity, and resistance. All the data were analysed with Review Manager (RevMan, Version 5.4, the Cochrane collaboration, 2020). The data were grouped by random effects, with a confidence interval of 95% (MD95%). Heterogeneity was assessed with an *I*-squared test. In the case that this value was above 50%, it would be classified as high, and the data would be relativized in the subsequent analysis. No publication bias study was conducted as we did not find more than 10 studies for any specific physical capacity [20].

## 3. Results

Of the five databases analysed, a total of 354 entries were considered eligible, according to the following distribution: SportDiscus (*n* = 31), PEDro (*n* = 21), PUBMED (*n* = 68), Web of Science Core Collection (*n* = 68), and B-ON (*n* = 166).

For this systematic review, 30 RCT studies were analysed. The remaining ones were non-randomized, and observational studies were included in the qualitative analysis in order to frame the practice and use of Pilates as a means of enhancing HA.

### 3.1. RCT Studies

The PEDro Scale assessment of the 30 studies resulted in 9 of low methodological quality, 14 of moderate quality, and 7 high-quality studies.

Table 1, Table 2, Table 3, Table 4 and Table 5 present the details of the studies analysed in this systematic review.

Of the six studies presented in Table 1, half did not report advantages of the Pilates method per se or in combination with other techniques and interventions [22,27]. The first did not find any evident benefit for trunk strength and balance, whereas the second did not find Pilates to be the most effective method in strength gains or in the transfer of these gains to the functional autonomy of the elderly. In contrast, one study shows a beneficial effect of Pilates in the functional autonomy of the elderly [24]. The remaining studies showed gains in flexibility [23], emotional health [25], balance, and reduction in the risk of falling [26].

The study of Gabizon et al. [28] is the third without evidence of a positive influence of Pilates. To the authors, this may be due to the fact that Pilates is not a specific method for the development of balance. On the other hand, there is the advantage of Pilates in reducing waist perimeter and BMI [30]. In the remaining studies, balance and confidence in balance [32] and improvements in walking ability and in fear of falling, with the corresponding reduction in the risk of falling, were highlighted by some authors [29,31]. Lower limb strength and functional autonomy were also reported as having benefited from Pilates [33].

Regarding this set of studies, lower limb strength improvements were reported [35,36,38]. Balance is also improved with Pilates, particularly for those with deficits in trunk control and trunk stability [37]. Jurakic et al. (2017) showed that Pilates is beneficial for elderly people with short-term memory deficits [34]. Finally, pulmonary function also improves [39].

Balance improvements, reduction in the risk of falling, increase in functional mobility, and postural stability are reported in this set of studies [42,44]. Additionally, improvements in pulmonary function are also reported [43]. Improvements in quality of life, satisfaction with life, and perception of health status were also reported [40,41,45], as well as improvements in functional autonomy [40] and sleep quality [41].

The studies presented a set of results considering the advantages of Pilates in balance [2,3,47] and strength [3,5,47]. One study revealed improvements in functional capacity, walking, and mobility [8]. The same authors also found beneficial effects of Pilates interventions in the cognitive dimension. Advantages in cardiorespiratory fitness were also reported [46].

In summary, 27 of the 30 studies analysed reported advantages of Pilates for the elderly. The areas where more advantages were reported were in static or dynamic balance [3,5,33,35,36,38,39,43,47]. Four studies showed benefits in total strength, three in lower limb strength, and two studies reported benefits in respiratory strength. Functional capacity and functional autonomy also tend to improve with Pilates, according to four studies [8,33,40,42]. The psychological and mental-health-related variables (e.g., perception of health, quality of life, satisfaction with life, emotional health, and sleep quality) also improved significantly with Pilates [25,40,41,45]. In two experimental studies, flexibility was improved [23,42], and two other studies showed improvement in aerobic resistance [38,46]. A decrease in BMI and waist perimeter was also reported [30]. Finally, gains in short-term memory were also reported in cognitively disabled people [34].

### 3.2. Observational or Non-Randomized Studies

Table 6 presents the details of the observational or non-randomized studies included in this review.

This section included 14 studies with similar benefits being reported, despite different methodologies used. Two studies show that Pilates is not the most effective intervention to control blood pressure, glycaemia [57], and the sensory regulation of static or dynamic balance [58]. The remaining studies, however, report gains in strength [48,53,59] and improvements in the functional capacity and mobility of the elderly [52,59,60]. Psychological variables, such as self-resilience [49], well-being [50], quality of life [54], or health-related psychological variables [9], benefited with Pilates interventions. Additionally, improvements in walking ability [59], haemodynamic behaviour [56], fall-risk reduction [55], and salivary S-IgA [51] were mentioned once.

## 4. Meta-Analysis

### 4.1. Balance

Static balance (One Leg Stance—OLS) was analysed in three studies [2,3,26] and dynamic balance gathered nine, divided between the Timed Up-and-Go Test (TUG) [2,3,26,29,38,42] and the Berg Balance Scale (BBS) [28,42,47], with a total sample of 196 individuals.

**Figure 2:**
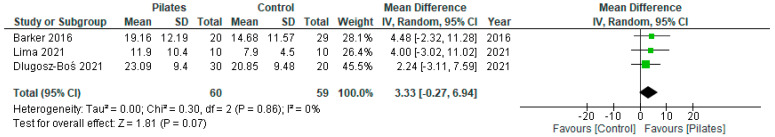
Meta-analysis of the comparison of intervention and control groups on OLS Test.

The results of the meta-analysis calculated for the OLS show a mean difference of 3.33 s between groups, without statistical significance (95% CI: [−0.27, 6.94]; *I*^2^: 0%) (Figure 2).

For the dynamic balance, when considering each test separately, we found a significant mean difference favouring the Pilates group compared to the control group of −0.60 s (95% CI: [−0.70, −0.49]; *I*^2^: 0%) in the TUG test. In the Berg Balance Scale (BBS) the results are not significant, despite the mean difference between groups also showing a better performance in the Pilates group (−1.46; 95% CI: [−3.06, 0.15]; *I*^2^: 33%).

**Figure 3:**
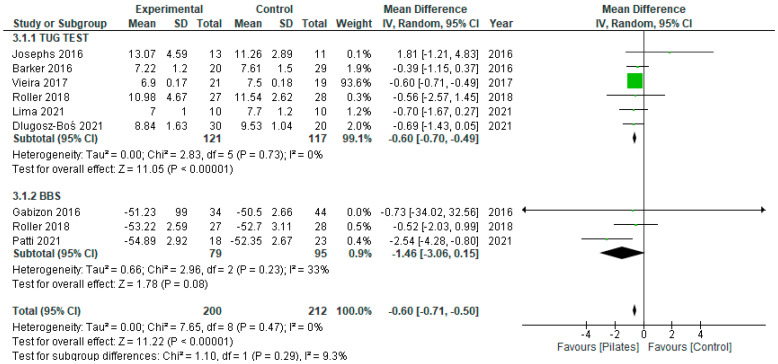
Meta-analysis of the comparison of intervention and control groups on dynamic balance.

For the global analysis of dynamic balance capacity, a significant difference in the mean difference was found between the Pilates and the control groups, with a value of −0.60 s (95% CI: [−0.71, −0.50]; *I*^2^: 0%) with an advantage for the first group (Figure 3).

**Figure 4:**
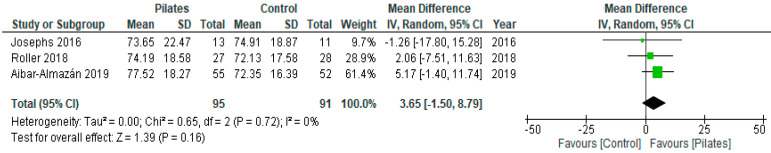
Meta-analysis of the comparison of intervention and control groups on ABC test.

The three studies that assessed confidence in balance using the ABC Scale [8,29,42] point to the absence of a significant mean difference between the groups (3.65; 95% CI: [−1.50, 8.79]; *I*^2^: 0%) (Figure 4).

### 4.2. Strength

**Figure 5:**
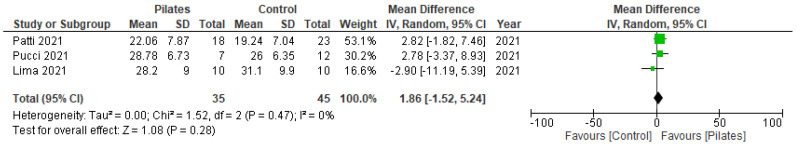
Meta-analysis of the comparison of intervention and control groups on handgrip test.

Regarding strength, three studies using handgrip strength were eligible [3,5,47]. The mean difference between both groups is not significant (1.86; 95% CI: [−1.52, 5.24]; *I*^2^: 0%) (Figure 5).

### 4.3. Aerobic Capacity and Aerobic Resistance

**Figure 6:**
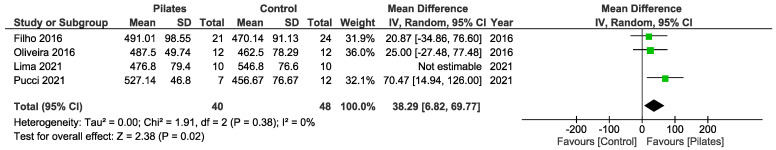
Meta-analysis of the comparison of intervention and control groups on 6 m walk test.

The 6 min walk test allows the assessment of aerobic capacity and aerobic resistance. From the studies analysed [3,5,24,27], statistically significant differences were found in the mean difference to complete the test: 38,29 m plus to the Pilates group (95% CI: [6.82, 69.77]; *I*^2^: 0%). The data from Lima et al., 2021, were not included in the meta-analysis due to their heterogeneity (Figure 6).

## 5. Discussion

The aim of this systematic revision is to collect and summarize the benefits of Pilates in the elderly population, within the current scientific production, assessing its contribution to Healthy Ageing (HA). In the first place, due to the state of the art and our findings, we could verify that the Pilates method has gained adepts over the last years. The increase in the elderly population is a fact and with it is the need to find processes that enable HA. Therefore, it is important to confirm the benefits of Pilates and scientifically validate them. The multiplication of the adaptations of this method, according to the current know-how or in order to meet the goals of the practice, has diversified the exercises and the reach of this method. A great example of this is the use of Pilates apparatuses that appear to point to differences between mat and apparatuses [61,62].

The use of springs and the consequent external load that they impose, along with the number of exercises made in an orthostatic position in these apparatuses may influence the results obtained. Similarly, the systematization difficulties and lack of consensus around the techniques and assessment instruments for the physical capabilities, among others, has resulted in a variability that creates difficulties in extrapolating and comparing the results and conclusions [63].

The results appear to show a robust tendency towards the benefits of Pilates for the elderly population. The analysis also points to a total absence of risks or contraindications of this method. Additionally, the benefits of this method are reinforced by the broad cultural and ethnic scope of the studies analysed, reducing any eventual contextual influence in the benefits presented.

Nevertheless, it is not clear that the Pilates method alone or in combination with other techniques and interventions [22,27] points to a robust tendency of the benefits of Pilates for the elder population. Still, it is important to emphasise the benefits of Pilates in the functional autonomy of the elderly [24], namely in flexibility [23], emotional health [25], and reduction in the risk of falling [26]. Moreover, there is an advantage of Pilates in reducing waist perimeter and BMI [30] and in improvements in walking ability [29,31,32]. Furthermore, the results also indicate that lower limb strength and functional autonomy were also reported as having benefited from Pilates [33].

Similarly, Jurakic et al. (2017) consider that Pilates has benefits for the elderly with short-term memory deficits [34] and that pulmonary function also improves [39]. In this context, improvements in quality of life, satisfaction with life, and perception of health status were also reported [40,41,45], as well as improvements in functional autonomy [40] and sleep quality [41]. Additionally, the results also showed the advantages of Pilates in balance [2,3,47], strength [3,5,47], improvements in functional capacity, walking and mobility [8], and in the cognitive dimension and cardiorespiratory function [46].

In a broader perspective, we emphasise that 27 of the 30 studies analysed reported the advantages of Pilates for the elderly. Standing out are the advantages in total strength, lower limb strength, functional capacity and functional autonomy [8,33,40,42]. Verified too are significant gains in psychological and mental health-related variables, in particular: perception of health, quality of life, satisfaction with life, emotional health [25,40,41,45], flexibility [23,42], aerobic resistance [38,46], waist perimeter [30]. Finally, advantages were also found for people with cognitive impairment [34].

Although the results point out that Pilates is not the most effective intervention to control blood pressure, glycaemia [57], and the sensory regulation of static or dynamic balance [58], we could not, however, fail to highlight the positive effects of Pilates on strength [48,53,59], improvements in the functional capacity and mobility of the elderly [52,59,60], and in psychological variables such as self-resilience [49] and well-being [50]. Furthermore, improvements in haemodynamic behaviour [56] and salivary S-IgA [51] were also mentioned in the systematic review of the studies.

Moreover, the meta-analysis shows some results that are consistent with the previous systematic reviews and meta-analysis regarding the benefits of Pilates for the elderly [10,15,16,63]. All the demonstrated advantages of this practice for the development of static and dynamic balance show some consistency and robustness in the results. Regarding aerobic capacity and resistance, the results are consistent with the meta-analysis of Bueno et al. (2018).

The main limitations of this review were the time limitation and the exclusion of information regarding other studies (e.g., books, magazines, or theses). Regarding the studies included in our analysis, the limitations are related to the clinical and methodological variability. Additionally, the high number of studies in which the control group did not suffer any type of intervention may condition the results, namely when calculating a more robust meta-analysis. Most of the studies had a sample composed mainly of women, which may also limit the analysis and interpretation of the results. Therefore, it is important to increase the research in this field, particularly with greater and more gender-balanced samples. Moreover, the protocols should be more standardized in order to better compare the results obtained. Future studies should focus on the analysis of the relationship between the cost and benefit of a Pilates intervention in the elderly population, to better understand how health costs can be minimized and to contribute to a multidisciplinary and generalized HA. Moreover, future systematic reviews may analyse which type of psychomotor responses are associated with the eventual neuromuscular benefits that may come from a Pilates intervention in the elderly population.

## 6. Conclusions

This review of studies shows a robust tendency towards the benefits of Pilates in physical capacity and in dynamic balance. The results also show that Pilates may be beneficial for the health of the elderly, contributing to HA that may slow down and fight the degenerative processes associated with senescence. It is also concluded that the efficacy of Pilates has been studied in various areas of HA and has proven to be affordable and safe for the majority of people, using just a mat on the floor. This way, clinicians, therapists, and exercise professionals that work with the elderly population may find in Pilates a viable strategy towards healthy ageing. The well-being improvements in the elderly are enabled by an association between the social and the physical components of practising Pilates, thus contributing to a healthier and more active ageing.

## Figures and Tables

**Table 1:** Structured summary of the studies included in the analysis (RCT).

*n*	Author	Title	Subjects/ Group	Objective	Intervention	Outcomes	Results	Conclusion(s)	PEDro Scale
1	Donath, L.; Roth, R.; Hürlimann, C.; Zahner, L.; Faude, O. (2016) [22]	Pilates vs. Balance Training in Health Community-Dwelling Seniors: a 3-arm, Randomized Controlled Trial	48 Pil 16 BAL 16 C 16 M/F	Examine the effects of traditional balance training methods vs. Pilates-based training in balance and trunk strength.	PIL: Mat Pilates BAL: Traditional balance training C: No intervention Duration: 8 weeks 2x week 66′	Freiburg Questionnaire Static Balance Dynamic Balance Perturbing Kneeling Trunk Strength	Substantial positive effects in favor of BAL compared to C were found for the Y balance score (right leg, effect size (d = 0.68; left leg, d = 0.56, trunk extension (d = 0.68 and single leg stance right leg, (d = 0.61;left leg, (d = 0.38. Dynamic (d = 0.32 and isometric (d = 0.15 trunk flexion revealed unclear effects. For the Y-balance score right leg, (d = 0.48, left leg, d = 0.75 and single leg stance right leg, (d = 0.61; left leg, d = 0.67, interestingly, BAL substantially exceeded PIL. PIL vs. CON revealed unclear effects for most parameters (0.05 < d < 0.36).	Mat-based Pilates training did not cause relevant adaptations in trunk strength and balance performance, whereas balance training substantially improved balance and trunk strength.	4/10
2	Oliveira, L.C.; Oliveira, R.G.; Pires-Oliveira, D.A. (2016) [23]	Comparison between static stretching and Pilates method on the flexibility of older women	32 16 + 16 F	To compare the effects of static stretching and Pilates on the flexibility of healthy older women, over the age of 60 years	Pilates: Static Stretching: Duration: 12 weeks 2x week 60′	Movements of the trunk (flexion and extension), hip flexion, and plantar and dorsiflexion of the ankle were performed before and after the intervention, using a fleximeter	The static stretching exercises improved the trunk flexion and hip flexion movements, while the Pilates improved all evaluated movements. However, over time, the groups presented differences only for the trunk extension movement	For some body segments, Pilates may be more effective for improving flexibility in older women compared to static stretching	7/10
3	Oliveira, L.C.; Pires-Oliveira, D.A.; Prado, R.A.; Oliveira, D.D.; Antônio, T.; Oliveira, R.F.; Oliveira, R.G. (2016) [24]	Effects of Pilates on postural balance and functional autonomy of the elderly: a randomized controlled trial	24 PA 12 C 12 M/F	To verify the effects of the Pilates method, based on the functional autonomy and postural balance in elderly women.	PA: Pilates apparatus SS: Static Stretching Duration: 8 weeks 2x week 60′	Battery of tests for functional autonomy of the elderly, the Group of Latin-American Development to the Maturity (GDLAM) protocol, six-minute walk test and static postural balance on a force platform.	The results showed significant results for GE in two tests of functional autonomy and the overall rate of functional autonomy (*p* < 0.05). Differences for the other tests were not found	The intervention protocol with Pilates, allowed the improvement of functional autonomy in elderly women, not having an effect on the six-minute walk test and the postural balance.	4/10
4	Ángeles, M.V.; Jiménez, J.M.; Sánchez, J.G.; Juan, F.R. (2016) [25]	Effects of a Pilates-based exercise program on mood states in older adults in Mexico	20 MP 10 C 10 M/F	Determine the effect of a Pilates-based conditioning program on the mood of the elderly.	MP: Pilates Mat C: No intervention Duration: 12 weeks 3x week 50′	Profile of Mood States (POMS)	Significative differences in pre- and post- measurements and between groups for Tension (*p* = 0.001), Fury (*p* = 0.030), Fatigue (*p* = 0.002) and total result (*p* < 0.0001).	Pilates improves some mood variables that may influence the emotional health of the elderly.	4/10
5	Barker, A.L.; Talevski, J.; Bohensky, M.A.; Brand, C.A.; Cameron, P.A.; Morello, R.T. (2016) [26]	Feasibility of Pilates exercise to decrease falls risk: a pilot randomized controlled trial in community-dwelling older people	43 PA 18 C 25 M/F	To evaluate the feasibility of Pilates exercise in older people to decrease falls risk and inform a larger trial.	PA: Pilates equipment C: Normal care Duration: 12 weeks 2x week 60′	Indicators of feasibility including: acceptability (recruitment, retention, intervention adherence and participant experience survey); safety (adverse events); and potential effectiveness (fall, fall injury and injurious fall rates; standing balance; lower limb strength; and flexibility) measured at 12 and 24 weeks.	Standing balance, lower-limb strength and flexibility improved in the Pilates group relative to the control group (*p* < 0.05). The rate of fall injuries at 24 weeks was 42% lower and injurious fall rates 64% lower in the Pilates group; however, it was not statistically significant (*p* = 0.347 and *p* = 0.136).	Pilates exercise is an enjoyable and acceptable form of exercise in community-dwelling older people at risk of falling. An appropriately designed Pilates exercise program appears to improve standing balance and reduce the risk of falls.	6/10
6	Filho, M.M.; Vianna, J.M.; Venturini, G.O.; Matos, D.G.; Ferreira, M.C. (2016) [27]	Assessment of different exercise programs on muscular strength and functional autonomy in the elderly	114 STG 22 GG 23 WAG24MP 21 C 24 F	Evaluate different types of physical exercises: strength training, gymnastics, water aerobics, and Pilates and a Control Group on elderly women’s muscular strength and functional autonomy.	STG: Multimuscular varied sessions GG:Multicomponent training (flexibility, strength, balance, agility) WAG:Aerobic and muscular workout MP:Pilates matwork C:No intervention Duration: 24 weeks 3x week 60′	Anthropometry Body Mass Index (BMI), Borg Rating of Perceived Exertion (RPE), Physical Fitness battery (Rikli and Jones, 1999).	All exercise modalities were efficient in increasing muscle strength and functional autonomy for the elderly participants in the proposed exercise programs (strength training, gymnastics, water aerobics, and Pilates), reinforcing the importance of an active lifestyle in this population.	Strength training overcame the other modalities about increasing muscle strength and transferring its physical capacity to functional autonomy.	4/10

**Table 2:** Structured summary of the studies included in the analysis (RCT).

*n*	Author	Title	Subjects/ Group	Objective	Intervention	Outcomes	Results	Conclusion(s)	PEDro Scale
7	Gabizon, H.; Press, Y.; Volkov, I.; Melzer, I. (2016) [28]	The Effects of Pilates Training on Balance Control and Self-Reported Health Status in Community-Dwelling Older Adults: A Randomized Controlled Trial	88 MP44 C 44 M/F	Evaluate the effects of a Pilates-based intervention balance and self-perception of health status.	MP: Floor Pilates C: No intervention Duration: 12 weeks 3x week	Standing upright postural stability, performance-based measures of balance, and self-reported health status were assessed in both groups at baseline and at the end of the intervention period.	Compared with the control group, the Pilates intervention did not improve postural stability, baseline functional measures of balance, or health status	The results suggest that because Pilates training is not task specific, it does not improve balance control or balance function in independent older adults.	7/10
8	Josephs, S; Pratt, M.L.; Meadows, E.C.; Thurmond, S.; Wagner, A. (2016) [29]	The effectiveness of Pilates on balance and falls in community-dwelling older adults	24 MP 13 C 11 M/F	Determine whether Pilates is more effective than traditional strength and balance exercises for improving balance measures, balance confidence, and reducing falls in community- dwelling older adults with fall risk.	MP: Pilates Matwork C: Traditional exercises Duration: 12 weeks 2x week 60′	Timed Up-and-Go test (TUG) Balance Test (FAB) Psychological Questionnaire (ABC)	There was significant improvement in the Fullerton Advanced Balance Scale for both the MP (mean difference = 6.31, *p* < 0.05) and the Control group (mean difference = 7.45, *p* = 0.01). The MP also showed significant improvement in the Activities-Specific Balance Confidence Scale (mean difference = 10.57, *p* = 0.008).	Both Pilates and traditional balance programs are effective at improving balance measures in community- dwelling older adults with fall risk, with the Pilates group showing improved balance confidence.	5/10
9	Pestana, M.S.; Netto, E.M.; Pestana, M.S.; Pestana, V.S.; Schinoni, M.I. (2016) [30]	Pilates versus resistance exercise on the serum levels of hs-CRP, in the abdominal circumference and body mass index (BMI) in elderly individuals	78 MP 39 RT 39 M/F	Compare the effects of Pilates vs. resistance training on seric levels of highly sensitive C-Protein (PCR-hs), waist perimeter (WP), and body mass index (BMI) in the elderly.	MP: Mat Pilates RT: Resistance training	PCR-hs WP BMI	Mat Pilates reached reductions in the seric levels of PCR-hs (Wilcoxon signed rank; z = −2.466, *p* = 0.01), on BMI (Wilcoxon signed rank; z = −3.295, *p* = 0.001), and in WP (Wilcoxon signed rank;. z = −3.398, *p* = 0.01). MP also obtained a significant reduction in the seric levels of PCR-hs and in the anthropometric measurements.	Pilates is more effective than resistance training in the reduction in waist perimeter and body mass index.	4/10
10	Roh, S; Yoon, S.Y.; Kim, J.N.; Lim, H.S. (2016) [31]	Effects of modified Pilates on variability of inter-joint coordination during walking in the elderly	20 MP 10 C 10 M/F	Examine the effects of an 8-week modified Pilates program on the vari- ability of inter-joint coordination in the elderly during walking.	MP: Pilates Matwork C: No intervention Duration: 12 weeks 2x week 60′	Three-dimensional motion analysis was performed on both groups to evaluate the effects of the Pilates exercise, calculating the continuous relative phase (CRP).	There was no significant difference in the joint variability of the ankle, knee, and hip joints between the groups, both before training and after training. There was a significant increase in the hip-knee deviation phase value in the MP and this increase was also significant when compared with that in the control group.	The 8-week modified Pilates exercise program can have a positive impact on the gait of elderly participants, potentially by enhancing neuromuscular adjustment, which may have positive implications for reducing their fall risk.	5/10
11	Badiei, M.; Shahboulaghi, F.M.; Hosseini, M.; Noroozi, M.; Nazari, S. (2017) [32]	Effect of Pilates Exercise on Fear of Falling in Iranian Elderly Women	44 MP 22 C 22 F	Determine the effect of Pilates exercise on Fear of Falling (FOF) among elderly women.	MP: Pilates Matwork C: Normal stretching training Duration: 8 weeks 3x week 60′	Data were gathered by using demographic questionnaire and Fall Efficacy Scale-International (FES-I).	FES-I scores in Pilates group improved from 32.90 to 22.18 (MD = 10.72) after the intervention. According to the independent *t*-test, there was a significant difference in the means of post-intervention FES-I scores between the two groups (*p* < 0.001). In the Pilates group, the effect size of intervention was much more than the control group (ES = 0.89).	Pilates training could decrease the FOF and may thus be implemented as an effective interventional method for fall prevention in elderly women.	6/10
12	Carvalho, F.T.; Mesquita, L.A.; Pereira, R.; Neto, O.P.; Zangaro, R.A. (2017) [33]	Pilates and Proprioceptive Neuromuscular Facilitation Methods Induce Similar Strength Gains but Different Neuromuscular Adaptations in Elderly Women	60 MP 20 PNF 20 C 20 F	To compare the influence of a training period with Pilates and Proprioceptive Neuromuscular Facilitation (PNF) methods on strength gains and motor control adaptations during voluntary contraction, applied to a group of elderly women.	MP: Pilates Matwork PNF: PNF training Duration: 4 weeks 3x week 50′	Isometric Force Acquisition EMG Measurement Fluctuations in Motor Output	One-way analysis of variance indicated no differences among groups for all variables (i.e., isometric force, force fluctuation, and force and EMG spectral features) at pre-training moment (*p* > 0.05). Isometric muscle force from knee extensors (KE) and flexors (KF) showed significant main effect for groups (F2,56 = 6.77, *p* = 0.002 from KE; F2,56 = 3.72, *p* = 0.03 from KF), for measure (F1,56 = 23.08, *p* < 0.0001 from KE; F1,56 = 21.23, *p* < 0.0001 from KF), and a significant Group × Measure interaction (F2,56 = 19.97, *p* < 0.0001 from KE; F2,56 = 6.65, *p* = 0.003 from KF).	These results support use of both Pilates and PNF methods to enhance lower limb muscle strength in older groups, which is very important for gait, postural stability, and performance of daily life activities.	5/10

**Table 3:** Structured summary of the studies included in the analysis (RCT).

*n*	Author	Title	Subjects/ Group	Objective	Intervention	Outcomes	Results	Conclusion(s)	PEDro Scale
13	Jurakic, Z.G.; Krizanic, V.; Sarabon, N.; Markovic, G. (2017) [34]	Effects of feedback-based balance and core resistance training vs. Pilates training on cognitive functions in older women with mild cognitive impairment: a pilot randomized controlled trial	28 MP 14 CRT14 F	Provide preliminary evidence on the effects of two types of non-aerobic training on cognitive functions in older women suffering from MCI (mild cognitive impairment).	MP:Pilates Matwork CRT:Core Resistance Training Duration: 8 weeks 3x week 60′	Assessing tool MCI: MoCA	CRT group obtained significant improvements in score of visuospatial/executive functions and orientation as well as global score compared with MP. Significant improvement in short-term memory-recall task was obtained only in the MP.	Non-aerobic training should be further explored as a beneficial intervention for older adults suffering from MCI.	5/10
14	Oliveira, L.C.; Pires-Oliveira, D.A.; Abucarub, A.C.; Oliveira, L.S.; Oliveira, R.G. (2017) [35]	Pilates increases isokinetic muscular strength of the elbow flexor and extensor muscles of older women: A randomized controlled clinical trial	30 PA 15 C 15 M/F	Verify the influence of Pilates in the isokinetic strength of the elbow extensors and flexors, as well as upper limb functionality	PA: Pilates apparatus C: Maintenance of routines Duration: 12 weeks 2x week 60′	Elbow extensor and flexor strength (dynamometer) Functional test (dress and undress a t-shirt)	In the intra-group comparison, the PA improved strength of the elbow extensors and the functionality of the upper limbs (*p* < 0.05). When comparing the post-intervention moment, the PA was superior to the C in all variables (*p* < 0.05), with a large effect size (d > 0.80).	It was observed that the Pilates method can contribute to improving the isokinetic muscular strength of the elbow flexors and extensors, as well as the functionality of the upper limbs.	8/10
15	Oliveira, L.C.; Oliveira, R.G.; Pires-Oliveira, D.A. (2017) [36]	Pilates increases isokinetic muscular strength of the knee flexor and extensor muscles of older women	32 PA 16 SS 16 F	Verify the influence of Pilates in the isokinetic strength of the knee extensors and flexors at 60°.	PA: Pilates apparatus SS: static stretching Duration: 12 weeks 2x week 60′	Knee extensor and flexor strength (dynamometer)	The SS presented a significant improvement (*p* < 0.01) in all tests performed, when comparing the pre- and post-intervention (intragroup) (Cohen’s d = 2.03 and 1.33 for the knee flexor and extensor muscles, respectively). Comparing the C and SS (intergroup), post-intervention, a significant improvement was observed (*p* < 0.01) in favor of the SS for all variables (Cohen’s d = 1.59 and 1.15 for the knee flexor and extensor muscles, respectively.	The results indicated that 12 weeks of Pilates increases the isokinetic muscular strength of the knee extensors and flexors in elderly women and can be considered for this purpose when prescribing physical exercise programs.	8/10
16	Sofianidis, G; Dimitriou, A.; Hatzitaki, V. (2017) [37]	A Comparative Study of the Effects of Pilates and Latin Dance on Static and Dynamic Balance in Older Adults	36 MP 12 LD 12 C 12 M/F	Compare the efficiency of Pilates intervention and Latin Dances in static and dynamic balance of the elderly.	MP: Mat Pilates LD: Latin Dances C: No intervention Duration: 12 weeks 2x week 60′	Center of Pressure (CoP) Trunk Angular Variability “Two-Leg Tandem Stance” with eyes open and closed “One Leg Stance” (OLS) with the eyes open Dynamic balance “Periodic balance” with and without guiding sound	The results of trunk swinging during the “Two-Leg Tandem Stance” with eyes closed, reduction on CoP displacement during the “One leg Stance” and the increase in trunk oscilation in the trunk swinging test for both intervention groups.	Both programs had a positive effect in the static and dynamic balance-related variables. LD appears to be better for people with rhythm perception and sensorimotor control. On the other hand, Pilates appears to be more effective for people with trunk control problems, as it improves core stability.	4/10
17	Vieira, N.D.; Testa, D.; Ruas, P.C.; Salvini, T.F.; Catai, A.M.; Melo, R.C. (2017) [38]	The effects of 12 weeks Pilates-inspired exercise training on functional performance in older women: A randomized clinical trial	40 MP 21 C 19 F	Investigate the effects of a 12-week Pilates-inspired program in the functional performance of elderly people in nursing homes.	MP:Mat Pilates C:No intervention Duration: 12 weeks 2x week 60′	(OLS) Timed Up-and-Go Test (TUG) five-time-sit-to-stand (STS) 6 min walk (6 MW)	After the intervention, significant differences were found in the time to complete the tasks: STS (*p* = 0.03) e 6 MW (*p* < 0.01). Only the MP improved the STS (*p* = 0.02) and the 6 MW test (*p* < 0.01).	Pilates-based exercises improve balance, lower limb strength and aerobic resistance in elderly ladies in nursing homes.	5/10
18	Alvarenga, G.M.; Charkovsky, S.A.; Santos, L.K.; Silva, M.B.; Tomaz, G.O.; Gamba, H.R. (2018) [39]	The influence of inspiratory muscle training combined with the Pilates method on lung function in elderly women: a randomized controlled trial	31 MP + TI 11 PA 11 C 9 F	Assess the influence of inspiratory muscle training combined with Pilates in the pulmonar function of elderly women.	MP + TI (Pilates Group + Inspiratory training) PA: Pilates Apparatus C: No intervention Duration: 2x week 45′ 10 weeks	Spirometry manuvacuometry 6 min test Curl-Up Test Pulmonary variables	Improvements in the strength of muscles in maximal inhalation and in pressure and power (*p* < 0.0001), in the muscular strength on maximal exhalation (*p* < 0.0014), in the performance of the 6 min test (*p* < 0.01), and in the Curl-Up test (*p* < 0.00001).	Pilates combined with technological equipment that allows the analysis, treatment, and training of the pulmonar function showed efficacy in this type of application.	5/10

**Table 4:** Structured summary of the studies included in the analysis (RCT).

*n*	Author	Title	Subjects/ Group	Objective	Intervention	Outcomes	Results	Conclusion(s)	PEDro Scale
19	Curi, V.S.; Haas, A.N.; Alves-Vilaça, J.; Fernandes, H.M. (2018) [40]	Effects of 16-weeks of Pilates on functional autonomy and life satisfaction among elderly women	61 MP 31 C 30 F	Determine the effects of Mat Pilates on the functional autonomy and life satisfaction of the elderly.	MP: Mat Pilates C: No intervention Duration: 16 weeks 2x week 60′	Rikli and Jones Protocol (2002) Satisfaction with life	Statistically significant differences were found in all parameters: lower limb strength and flexibility, upper limb strength and flexibility, dynamic balance, aerobic resistance, and satisfaction with life.	Functional autonomy and satisfaction with life were improved with the intervention, suggesting that Pilates helps in active ageing.	5/10
20	Curi, V.S.; Haas, A.N.; Alves-Vilaça, J.; Fernandes, H.M. (2018) [41]	Effects of 16-weeks of Pilates on health perception and sleep quality among elderly women	61 MP 31 C 30 F	Determine the effects of Mat Pilates on the perception and sleep quality of elderly.	MP: Mat Pilates C: No intervention Duration: 16 weeks 2x week 60′	General Health Questionnaire (GHQ−12) Pittsburgh Sleep Quality Index (PSQI-BR)	Statistically significant differences were found in the following parameters: GHQ−12 total score (*p* < 0.001, η^2^ = 0.19) Depression sub-scale (*p* < 0.002, η^2^ = 0.15) Social disfunction Sub-scale (*p* < 0.001, η^2^ = 0.18) PSQI-BR total score (*p* < 0.017, η^2^ = 0.09) sleep latency (*p* < 0.023, η^2^ = 0.09) and Use of medication sub-scale (*p* < 0.019, η^2^ = 0.09)	Health perception and other sleep quality parameters improved with Pilates.	5/10
21	Roller, M.; Kachingwe, A.; Beling, J.; Ickes, D.; Cabot, A.; Shrier, G. (2018) [42]	Pilates Reformer exercises for fall risk reduction in older adults: A randomized controlled trial	57 PA 27 C 28 M/F	Investigate the effects of Pilates using a Reformer in the fall risk, balance and mobility, self-efficacy, and active range of motion.	PA: Pilates in the Reformer C: no intervention Duration: 10 weeks 1x week 45′	Balance: “Sensory Organization Test and Adaptation Test” (SOT) and (ADT) “Timed Up-and-Go Test” (TUG) “Berg Balance Scale” (BBS) “Ten-meter walk test” (10 MTW) “Activities-specific Balance Confidence Scale” (ABC) Active range of motion: Straight Leg Raise (SLR) Hip Extension (HE) Ankle Dorsiflexion (AD)	An interaction between group and time in TUG, BBS, 10 MWT and SLR, HE, and AD. With time, PA significantly improved in all balance measurements (*p* ≤ 0.005). Improvements in active range of motion were found after the intervention for SLR (left) and AD (right).	Pilates Reformer done once per week for 10 weeks resulted in fall-risk reduction and significant improvements in the dynamic and static balance, functional mobility, self efficacy in balance and in lower limb range of motion. Pilates Reformer exercises are more effective than no exercises for improvements in the hip and ankle range of motion≥.	6/10
22	Tozim, B.M.; Navega, M.T. (2018) [43]	Effect of Pilates method on inspiratory and expiratory muscle strength in the elderly	31 MP 14 C 17 F	Analyse the influence of Pilates on respiratory strength in the elderly.	MP: Pilates matwork C: Educational sessions Duration: 8 weeks 2x week 60′	Maximal inspiratory Force (Pimax) Maximal expiratory Force (Pemax)	Significant differences were found with average effect size for the strength of the expiratory muscles in the MP when comparing pre- and post- (*p* < 0.05) intervention (69.71 ± 25.48 e 85.23 ± 22.21, respectively).	Pilates is effective in the improvement of expiratory muscle strength and presents a positive effect on the increase in the inspiratory muscle strength.	5/10
23	Aibar-Almazán, A.; Martínez-Amat, A.; Cruz-Díaz, D.; Torre-Cruz, M.J.; Jiménez-Garcia, J.D.; Zagalaz-Anula, N; Pérez-Herrezuelo, I.; Hita-Contreras, F. (2019) [44]	Effects of Pilates on fall risk factors in community-dwelling elderly women: a randomized, controlled trial	110 MP 55 C 52 F	Analyse the effect of Pilates on the confidence of balance, fear of falling, and postural control.	MP: 1x week 60′ C: No intervention Duration.12 weeks	Falls Efficacy Scale-International (FES-I) Activity-specific balance confidence scale (ABS) Stabilizing platform	MP presents higher values in the confidence of balance when compared with the C (77.52 ± 18.27 vs. 72.35 ± 16.39, Cohen’s *d* = 0.030), as well as in fear of falling (22.07 ± 5.73 vs. 27.9 ± 6.95, Cohen’s *d* = 0.041). MP significantly improved the speed and antero-posterior movements of the center of pressure with both open and closed eyes (Cohen’s *d* = 0.44 e 0.35, respectively).	A 12-week Pilates intervention is beneficial for the confidence in balance, fear of falling, and postural stability in elderly women.	8/10
24	Liposcki, D.B.; Nagata, I.S.; Silvano, G.A.; Zanella, K.; Schneider, R.H. (2019) [45]	Influence of a Pilates exercise program on the quality of life of sedentary elderly people: A randomized clinical trial	24 PA 9 C 11 F	Assess the influence of Pilates on quality of life of sedentary elderly people.	PA: Pilates Group: Mat and apparatus C (Control): Normal routine Duration: 6 months 2 x week 30′	QOL SF−36	MP improved in 7 of the 8 domains of this study: functional capacity(91.6 ± 14.3 vs. 62.6 ± 24.4; *p* ≤ 0.01); physical aspect (92.7 ± 14.8 vs. 52.2 ± 43.9; *p* = 0.03); pain (95.7 ± 6.9 vs. 52.2 ± 17.5; *p* ≤ 0.01); General health status (89.4 ± 11.2 vs. 76.7 ± 16.3; *p* = 0.04); Vitality (85.5 ± 13.5 vs. 70.0 ± 14.9; *p* = 0.04); Social aspects (97.2 ± 8.3 vs. 77.9 ± 23.9; *p* = 0.03); and mental health (77.52 ± 18.27 vs. 72.35 ± 16.39; *p* = 0.05).	Results show that implementing a Pilates program may improve the quality of life of sedentary elderly people.	4/10

**Table 5:** Structured summary of the studies included in the analysis (RCT).

*n*	Author	Title	Subjects/ Group	Objective	Intervention	Outcomes	Results	Conclusion(s)	PEDro Scale
25	García-Garro, p.A.; Hita-Contreras, F.; Martínez-Amat, A.; Achalandabaso-Ochoa, A.; Jiménez-García, J.D.; Cruz-Díaz, D.; Aibar-Almazán, A. (2020) [8]	Effectiveness of A Pilates Training Program on Cognitive and Functional Abilities in Postmenopausal Women	110 MP 55 C 55 F	To determine the effects of a Pilates exercises program on the cognitive and physical functioning of older Spanish women.	MP: Pilates-based matwork C: No intervention Duration: 12 weeks 2x week 60′	Global cognitive function (Mini-Mental State Examination), verbal fluency (Isaacs test), executive function (Trail Making Test), functional flexibility (Back Scratch Test and Chair Sit-and-Reach Test), and lower-body strength (30 s Chair-Stand Test)	The main findings of this study suggest that women in the MP (within-group differences) experienced improvements across all the variables examined except for global cognitive function. When compared with the C (between-group differences), our analysis revealed significant benefits in the MP for all measures except for global cognitive function and functional flexibility (Back Scratch Test).	The results suggest that Pilates has the potential to improve both cognitive and functional abilities among Spanish women aged 60 years and over.	8/10
26	Lima, M.; Silva, B.; Rocha-Rodrigues, S.; Bezerra, P. (2021) [3]	The impact of an 8-week Pilates-based physical training program on functional mobility: data from a septuagenarian group	20 MP 10 C 10 M/F	Assess the effects of a Pilates program on functional mobility and strength of elderly in nursing homes.	MP: Mat Pilates C: No intervention Duration: 8 weeks 2x week 60′	Anthropometric measurements: Weight, height, BMI, waist perimeter, thigh perimeter Strength tests (upper and lower limbs) 6 min walk test Balance test (OLS with open and closed eyes) Functional Mobility (TUG)	MP obtained higher results for lower limb strength (*p* = 0.013; *d* = 0.56) and for the 6 min walk test (*p* = 0.04; *d* = 0.45) when compared to the Control Group (C). MP also obtained better results in the “OLS” and “TUG” tests. Significant correlations were found between strength and cardiorespiratory fitness (*p* < 0.01, r = 0.62), between cardiorespiratory fitness, and “OLS”, eyes closed and both lower limbs (*p* = 0.04, r = 0.45; *p* = 0.05, r = 0.45), respectively.	8 weeks of Pilates improved strength and functional mobility in elderly people living in nursing homes.	6/10
27	Buttelli, A.K.; Costa, R.R.; Farinha, J.B.; Fagundes, A.O.; Vieira, A.F.; Barroso, B.M.; Bracht, C.G.; Coconcelli, L.; Reichert, T.; Rocha, V.B.; Kruel, L.M. (2021) [46]	Pilates training improves aerobic capacity, but not lipid or lipoprotein levels in elderly women with dyslipidemia: A controlled trial	26 MP 20 C 6	To verify the effects of Pilates training on total cholesterol (TC), triglycerides (TG), low-density lipoprotein (LDL), high-density lipoprotein (HDL), glucose, and C-reactive protein (CRP) levels, as well as on functionality of postmenopausal women with dyslipidemia.	MP: Pilates-based matwork C: No intervention Duration: 10 weeks 2 to 4 x week 45 to 55′	Biochemical analyses and functionality parameters were measured before and after the 10 weeks.	No significant differences were observed in TC, TG, LDL, and HDL for both groups. Regarding glucose and CRP levels, significant reductions were observed in both groups after the intervention period. In functional parameters, both groups significantly increased their 30 s chair stand test values. On the other hand, only the Pilates group presented significant increments in the 6 min walk test (*p* < 0.05).	Pilates training did not change lipid or lipoprotein levels, but improved the cardiorespiratory fitness of elderly women with dyslipidemia,	4/10
28	Dlugosz-Boś, M.; Filar-Mierzwa, K.; Stawarz, R.; Ścislowska-Czarnecka, A.; Jankowicz-Szymańska, A.; Bac, A. (2021) [2]	Effect of Three Months Pilates Training on Balance and Fall Risk in Older Women	50 MP 30 C 20 F	To assess the effect of Pilates exercises on balance and fall risk in older women.	MP: Pilates-based matwork C: No intervention Duration: 12 weeks 2x week 45′	Timed Up-and-Go (TUG) OLST Freestep baropodometric platform and Biosway platform	After intervention, significantly decreased values of the surface of the ellipse (*p* = 0.0037) and mean values of velocity (*p* = 0.0262) for the right foot in the experimental group were observed. The Limits of Stability (LoS) test (*p* = 0.005) and the Modified Clinical Test of Sensory Interaction on Balance (m-CTSIB) performed on an unstable surface with eyes closed (*p* = 0.0409) indicated statistically significant changes in the experimental group.	Pilates training affected the participants’ balance by improving LoS and reducing fall risk.	4/10
29	Patti, A.; Zangla, D.; Sahin, F.N.; Cataldi, S.; Lavanco, G.; Palma, A.; Fischietti, F. (2021) [47]	Physical exercise and prevention of falls. Effects of a Pilates training method compared with a general physical activity program A randomized controlled trial	41 MP 18 PAG 23 M/F	To compare a general physical activity program for the elderly with a Pilates program to evaluate the effects on balance and on reducing the risk of falling.	MP: Pilates-based matwork PAG: Non-specific program of physical activity Duration: 13 weeks 3x week 50′	Handgrip test Berg balance scale test (BBS) and posturographic analysis	Spearman correlation coefficient showed correlations between the following parameters: BBS versus handgrip test (r = 0.68); BBS versus ellipse surface area (r = 0.75). There were significant differences between groups after the exercise program: both groups showed an improvement in performance but the MP recorded significantly better results than the PAG.	Physical activity improves both balance and strength. However, the data show that Pilates has a greater effect on these physical abilities than a general physical activity program.	7/10
30	Pucci G.F.; Neves, E.B.; Santana, F.S.; Neves, D.A.; Saavedra, F.F. (2021) [5]	Comparative analysis of pilates and resistance trianing in physical fitness of the elderly	25 MP 7 TR 6 GLC 12 F	Assess the effects of two exercise interventions (resistance training and Pilates) in the elderly.	MP: Mat Pilates TR: Resistance training GLC: games, memory exercises, motor coordination exercises. Duration: 24 Weeks 2x Week 60′	Senior Fitness Test—Test battery for physical fitness in the elderly (strength, aerobic resistance, flexibility, dynamic balance, agility and Body Mass Index)	There was a significant increase for the MP and TR groups in the forearm flexion test pre- and post-intervention. The TR group also showed an increasing the handgrip test, particularly between week 12 and 24.	Pilates and resistance training contributed to a significant increase in the strength of elderly.	5/10

**Table 6:** Structured summary of observational or non-randomized studies included in the review.

*n*	Author	Title	Subjects/ Group	Objective	Intervention	Outcomes	Results	Conclusion(s)
1	Queiroz, L.S.; Bertolini, S.G.; Benneman, R.M.; Silva, E.S. (2016) [48]	The effect Mat Pilates practice on muscle mass in elderly women	MPA 43 F	To verify that the Mat Pilates practice increases muscle mass in elderly women.	MP: Pilates Mat and Apparatus Duration: 12 weeks 3x week 40′	Evaluation of arm muscle area before and after intervention Tricep skinfold	Statistically significant difference was observed (*p* < 0.002) between the average value of the arm muscle area, before (35.56 cm^2^) and after the exercises. (42.72 cm^2^)	Mat Pilates program generates positive effect on increasing the muscle mass of the elderly.
2	Roh, S.Y. (2016) [49]	Effects of a 16-week Pilates exercise program on the ego resiliency and depression in elderly women	148 F	Study the effects of a 16-week Pilates program on self-resilience and depression of elderly women.	Mat Pilates Duration: 16 weeks’ 3x week 50′	Self-Resilience questionnaire (Klohnen 1996) “Geriatric Depression Scale-Short Form” (Sheikh and Yesavage 1986)	All sub variables of self-resilience obtained a significantly higher score after the intervention: self-confidence (t = 7770, *p* < 0.001), efficiency of communication (t = 2690, *p* < 0.001), Optimistic trait (t = 1996, *p* < 0.05) and anger management (t = 4525, *p* < 0.001). Regarding the Geriatric Depression Scale, the values were significantly lower after the intervention (t = −6506, *p* < 0.001)	Pilates helped participants improve their self-resilience and relieved their levels of depression.
3	Roh, S.Y. (2016) [50]	Effects of a 12-week Pilates exercise program on wellness in elderly	88	Examine the efficiency of a 12-week Pilates program for the elderly.	Mat Pilates Duration: 12 weeks 3x week 50′	Well-being Questionnaire	After 12 weeks of practice, participants felt significant improvements in the various domains in the questionnaire: physical, (t = 2762, *p* < 0.01), social (t = 3362, *p* < 0.001), spiritual (t = 2307, *p* < 0.05), and emotional well-being (t = 2489, *p* < 0.05).	Pilates helped improve the well-being of the elderly.
4	Hwang, Y.; Park, J.; and Lim, K. (2016) [51]	Effects of Pilates Exercise on Salivary Secretory Immunoglobulin A Levels in Older Women	12 MP 6 C 6 F	Examine the effects of a Pilates program on the immune function of older women.	MP: Pilates Matwork C: No workout Duration: 12 weeks 3x week 50′	Aerobic Power (Astrand) Salivary secretions Questionnaire about colds and respiratory ilnessess	Salivary flow was significantly higher in the MP than in the C. After the acute high-intensity exercises were performed following the three-month Pilates exercise program, the salivary flow rate was significantly higher at all time points. The S-IgA secretion rate significantly increased 30 min after acute high-intensity exercise performed following the three-month Pilates exercise program.	Regular participation in a moderate-intensity Pilates exercise program can increase salivary flow rate and S-IgA secretion in older women.
5	Bertoli, J.; Biduski, G.M.; Freitas, C.R. (2017) [52]	Six weeks of Mat Pilates training are enough to improve functional capacity in elderly women	MP 18 F	Assess the effects of Mat Pilates on the functional capacity of elderly women.	MP: Mat Pilates Duration: 6 weeks 3x week 60′	Timed Up-and-Go Test Time Up Stairs Time Down Stairs 30 s Chair Stand Chair sit-and-reach Back Scratch	All tests showed a significant improvement after the intervention: Timed Up-and-Go Test 5.28 ± 0.11 and 4.86 ± 0.09; *p* = 0.009 Timed Up Stairs 3.04 ± 0.10 and 2.71 ± 0.07; *p* = 0.009 Timed Down Stairs 2.92 ± 0.14 and 2.56 ± 0.15; *p* = 0.001 30 s Chair Stand 15 ± 0.05 and 16 ± 0.52; *p* = 0.001 Chair sit-and-reach 7.14 ± 2.80 and 15.16 ± 3.05; *p* = 0.001 Back Scratch −2.33 ± 2.79 and −0.82 ± 2.56; *p* = 0.001.	There was a significant improvement of the functional capacity of the elderly women after the intervention.
6	Bertoli, J.; Pupo, J.; Vaz, M.A.; Detanico, D.; Biduski, G.M.; Freitas, C.R. (2018) [53]	Effects of Mat Pilates on hip and knee isokinetic torque parameters in elderly women	MP 42 F	Analyse the effects of Mat Pilates on the isokinetic strength of the lower limbs in elderly women.	MP: Mat Pilates Duration: 12 weeks 3x week 60′	Isokinetic Dynamometer	Mat Pilates produces improvements in knee flexors strength and in hip flexors, and extersors maximum strength (6 weeks). Improvements in maximum strength and mechanical work of the knee extensors and hip flexors and extensors were also verified after the 12-week intervention.	Pilates training periodization induces significant improvements in isokinetic strength.
7	Mello, N.F.; Costa, D.L.; Vasconcellos, S.V.; Lensen, C.M.; Corazza, S.T. (2018) [54]	Contemporary Pilates in physical fitness, cognition and quality of life in the elderly	16 MP 8 C 8 M/F	To evaluate the effects of the contemporary Pilates method on the physical fitness, cognition, and quality of life of the elderly.	MP: Pilates Matwork C: No intervention Duration: 15 weeks 2x week 60′	The Senior Fitness Test battery (SFT), the Vienna Test System and the EUROHIS QOL−8 quality-of-life questionnaire were used.	Significant difference (*p* < 0.05) was observed in the variables: lower and upper extremity flexibility test (*p* = 0.007), agility (*p* = 0.001) and dynamic balance (*p* = 0.001), aerobic endurance test (*p* = 0.001) and Attention and concentration test time (*p* = 0.047)	The contemporary Pilates method improves the health of the elderly, thereby helping to promote quality of life.
8	Scherf, B.G.; Guadagnin, E.C.; Tier, C.G.; De Almeida Dias, S.L. (2019) [55]	Effect of a Mat Pilates protocol on fall risk in elderly women.	MP 16 F	To verify if a short-term Mat Pilates protocol is effective in reducing fall risk in elderly women.	MP: Mat Pilates Duration: 5 weeks 2x week 60′	Sharp Romberg Test Berg Balance Scale (BBS) 30 s sit-to-stand testTimed Up-and-Go test (TUG) Gait Kinematic assessment	Significant improvements in strength, static, and dynamic balance, functional mobility, gait speed, and step length were verified.	A short term Mat Pilates protocol, may be an efficient alternative to reduce fall risk in the elderly population.
9	Bueno, J.A.; Alves, R.G.; Smoralek, A.C.; Junior, T.S. (2019) [56]	Haemodynamic and perceptual behaviour in elderly women subjected to one session of different Pilates protocols.	19 PA 10 MP 9 F	Verify the haemodynamic behaviour and compare the physiological response and safety of two Pilates protocols (mat and apparatus) in elderly women.	PA: Pilates Apparatus MP: Mat Pilates	Heart Rate (HR) Blood Pressure (BP) Perceived Exhersion (PE) Double product, calculated as follows: HR.mmHg.bpm Scale of Perceived Exhertion (OMNI-RES)	Diastolic pressure during recovery after 60 min increased 10 bpm (*p* < 0.04), as well as lower HR for the PA during recovery HR (10 min) (*p* < 0.03) and post-recovery (30 min) (*p* < 0.03). For both groups, the results of the acute session did not show abnormalities in the double product, with minimum PA and MP values of 9.820 and 8.740 mmHg.bpm and maximum values of 13.824 and 11.771 mmHg.bpm, respectively. The average was very close between both groups: PA 11.416 and MP 10.105 mmHg.bpm, respectively.	Both protocols appear to be safe and efficient for normotensive elderly women.
10	Nascimento, M.M.; Pereira, L.D.; Júnior, E.C.; Castro, H.G.; Appell, H.J. (2019) [57]	Assess the exteroceptive and interoceptive regulation of body balance in active elderly women	32 MP1 8 MP2 8 MP3 8 MP4 8 F	To evaluate the balance performance of a group of physically active elderly women, with emphasis on the regulation of the visual, vestibular, and somatosensory systems of the static and dynamic balance, as well as their performance in gait tests and flexibility.	MP1: Pilates matwork (60–64 years old) MP2: Pilates matwork (65–69 years old) MP3: Pilates matwork (70–74 years old) MP4: Pilates matwork (75–79 years old) Duration: 2x week 60′	Berg Balance Scale (BBS) Timed Up-and-Go Test (TUG) Functional Reach Test (TAF), and Body Balance Test (TEC)	BBS attained a conserved equilibrium [F(3.29) = 1.766, *p* ≥ 0.50], the TUG for preserved functional independence [F(3.29) = 0.418; *p* ≥ 0.50], and TAF without risk of falls. [F(3,20) = 2228; *p* ≥ 0.50]. The TEC identified deficit of interoceptive regulation of balance for all ages and compromise of interoceptive regulation of dynamic balance for septuagenarians [F(3,29) = 0.301; *p* ≥ 0.50]. A moderate correlation was observed between BBS-TEC (r = 0.416, *p* = 0.018), TEC-TUG (r = −345, *p* = 0.013) and negative between BBS-TUG (r = −0.427, *p* = 0.013). The TAF did not prove to be equivalent to the other instruments.	Sexagenarian and septuagenarian women, regular physical exercise practitioners, despite indicating good performance in tests of balance, gait and flexibility, presented impairment of the systems of sensorial regulation of the static and dynamic balance
11	Machado, O.S.; Campos, S.P.; Killian, L.F.; Machado, G.C.; Gianolla, F. (2020) [58]	Effect of a single exercise session on blood glucose and blood pressure in elderly	30 AE 6 RT 6 F 6 MP 6 C 6 M/F	To analyse the acute effect of exercise on post-exercise hypotension (PEH) and blood glucose in the elderly.	AE (Aquatic Exercise) RT (Resist. Training) F (Functional/ Weight-bearing Training) MP (Pilates) C (No intervention) Duration: 26 weeks 3x week 60′	Blood glucose was recorded 15 min before the beginning of the exercise session and 30 min after the end of it	RT (systolic PEH and blood glucose) and the AE (diastolic PEH) were able to promote the most significant reductions when compared to other modalities.	RT and AE were preferentially recommended for the elderly population with characteristics close to the sample used in this research.
12	Choi, W.; Joo, Y.; Lee, S. (2021) [59]	Pilates exercise focused on ankle movements for improving gait ability in older women	MP 22 F	To determine whether Pilates exercise can improve gait, muscle strength, and mobility in community- dwelling older women	MP: Pilates matwork	Weight BMI Skeletal Muscle Mass Body Fat Percentage Waist-Hip ratio Gait: Speed Cadence Step length Stride length Bilateral Handgrip Strength 30 s chair sit-to-stand test Abdominal Strength Manual Muscle test of ankle dorsiflexors and plantar flexors	Significant improvements were observed in the spatial gait parameters, muscle strength, and range of motion of ankle (*p* < 0.05).	Pilates exercise is beneficial exercise to improve gait, muscle strength, and mobility in community-dwelling older women.
13	Villarreal-Angeles, M.A.; Moncada-Jiménez, J; Ruiz-Juan, F. (2021) [9]	Improvement of psychological variables through Pilates in the elderly	20 MP 10 C 10 F	Determine the effect of a Pilates program on psychological variables in the elderly.	MP: Mat Pilates C: No intervention Duration: 12 Weeks 3x week 50′	WHOQOL−100 Questionnaire	A significant interaction (*p* < 0.05) between pre- and post-intervention, and between groups (MP e C) was verified in the following dimensions: physical health, social relationships, and psychological aspects.	A 12-week Pilates training program allows the improvement of psychological variables relevant for the elderly person’s good health.
14	Mueller, D.; Redkva, P.E.; Borba, E.F.; Barbosa, S.C.; Krause, M.P.; Silva, S.G. (2021) [60]	Effect of Mat vs. apparatus pilates training on the functional capacity of elderly women	48 PA 15 MP 16 C 17 F	Compare mat Pilates and apparatus Pilates on the functional capacilty of elderly women.	PA: Apparatus Pilates MP: Mat Pilates C:No Pilates Duration: 8 Weeks 2x week 50′	Senior Fitness Tests (SFT)	Both Pilates interventions obtained improvements in strength and flexibility of lower and upper limbs, as well as aerobic fitness and agility.	Apparatus and mat Pilates offer similar significant improvements in the functional capacity of elderly women.

## Data Availability

The data presented in this study are available on request by the corresponding author.
